# Preoperative subjective assessment of disease‐specific quality of life significantly influenced the likelihood of achieving the minimal clinically important difference after surgical stabilization for recurrent lateral patellar instability

**DOI:** 10.1002/ksa.12319

**Published:** 2024-06-21

**Authors:** Danko Dan Milinkovic, Sebastian Schmidt, Julian Fluegel, Sebastian Gebhardt, Felix Zimmermann, Peter Balcarek

**Affiliations:** ^1^ Center for Musculoskeletal Surgery Charité‐University Medicine Berlin Berlin Germany; ^2^ Clinic for Orthopedic surgery, Vidia Kliniken Karlsruhe Germany; ^3^ Arcus Sportklinik Pforzheim Germany; ^4^ Center for Orthopaedics, Trauma Surgery and Rehabilitation University of Greifswald Greifswald Germany; ^5^ Berufsgenossenschaftliche Unfallklinik Ludwigshafen Ludwigshafen am Rhein Germany; ^6^ Department of Trauma Surgery, Orthopaedics, and Plastic Surgery University Medicine Göttingen Göttingen Germany

**Keywords:** age, knee function, medial patellofemoral ligament (MPFL), minimal clinically important difference (MCID), patellar instability, patient‐reported outcomes (PROMs)

## Abstract

**Purpose:**

To evaluate which factors exert a predictive value for not reaching the minimal clinically important difference (MCID) in patients who underwent a tailored operative treatment for recurrent lateral patellar dislocation (RLPD).

**Methods:**

A total of 237 patients (male/female 71/166; 22.4 ± 6.8 years) were included. The Banff Patellofemoral Instability Instrument 2.0 (BPII 2.0) and subjective rating of knee function and pain (numeric analogue scale [NAS]; 0–10) were used to evaluate patients' outcomes from pre‐ to postoperatively. Gender, age at the time of surgery, body mass index (BMI), nicotine abuse, psychiatric diseases, cartilage status and pathoanatomic risk factors were evaluated as potential predictors for achieving the MCID using univariate logistic regression analysis.

**Results:**

The MCID for the BPII 2.0 was calculated at 9.5 points. Although the BPII 2.0 and NAS for knee function and pain improved significantly in the total cohort from pre‐ to postoperatively (all *p* < 0.001), 29 patients did not reach the MCID at the final follow‐up. The analysis yielded that only the preoperative NAS for function and BPII 2.0 score values were significant predictors for reaching the MCID postoperatively. The optimal threshold was calculated at 7 (NAS function) and 65.2 points (BPII 2.0). Age at the time of surgery should be considered for patients with a preoperative BPII 2.0 score >62.5.

**Conclusion:**

The probability of reaching BPII 2.0 MCID postoperatively depends only on the preoperative BPII 2.0 value and subjective rating of knee function, as well as age at the time of surgery for patients undergoing surgical treatment of RLPD. Here, presented results can assist clinicians in advising and presenting patients with potential outcomes following treatment for this often complex and multifactorial pathology.

**Level of Evidence:**

Level III.

**Abbreviations**
ACL‐R: anterior cruciate ligament reconstruction
AMADEUS: Area Measurement and Depth & Underlying Structures
BMI: body mass index
BPII 2.0: Banff Patellofemoral Instability Instrument 2.0
CD: Caton–Deschamps index
DFO: distal femoral osteotomy
HRQoL: health‐related quality of life
ICRS: International Cartilage Regeneration & Joint Preservation Society score
LR: lateral retinaculum
MCID: minimal clinically important difference
MPFL: medial patellofemoral ligament
MPFL‐R: medial patellofemoral ligament reconstruction
NAS: numerical analogue scale
PASS: patient acceptable symptomatic state
PF: patellofemoral
PROM(s): patient‐reported outcome measure(s)
QoL: quality of life
RLPD: recurrent lateral patellar dislocation
SCB: substantial clinical benefit
SF‐36: short form 36
TD: trochlear dysplasia
TP: trochleoplasty
TTO: tibial tuberosity osteotomy
TT‐PCL: tibial tubercle–posterior cruciate ligament
TT‐TG: tibial tubercle–trochlear groove

## INTRODUCTION

Medial patellofemoral ligament reconstruction (MPFL‐R) has become the cornerstone of surgical treatment for recurrent lateral patellar dislocation (RLPD), primarily owing to the satisfactory clinical outcomes, low recurrence rates, and overall high patient satisfaction following these procedures in the majority of reported cases [3, 4, 13, 40, 43, 44, 45]. However, several demographic, clinical and pathoanatomic factors have been outlined as predisposing to RLPD as well [2, 7, 16, 28]. These include younger age, history of contralateral patellar dislocation, skeletal immaturity [4, 17, 21, 28, 41, 43, 44], and a number and severity of osseous abnormalities, such as patella alta, trochlear dysplasia, lateralized tibial tubercle, valgus malalignment and increased femoral anteversion and external rotational malalignment of the tibia [4, 16, 18, 19, 35, 50, 51]. Given this high heterogeneity of various contributing parameters, establishing patient selection criteria and determining the precise indications for supplementary procedures in conjunction with MPFL‐R remains challenging [4, 18, 35, 47, 48, 51]. Furthermore, within this intricate constellation of underlying factors, understanding their individual and combined impacts on patellar stability, as well as on patient‐reported outcome measures (PROMs) prior to and after surgical stabilization procedures, remains inconclusive. This ambiguity is highlighted by varying and, at times, considerably diverging data found across different studies in the available literature [1, 2, 3, 4, 27, 28, 29, 50, 52]. However, irrespective of the clinical and pathoanatomic risk profiles, as well as surgical treatment strategies, previous data indicate that the majority of patients treated for RLPD experience notable improvements concerning patellar stability and early symptom‐free return to everyday and athletic activities [1, 18, 19, 21, 45, 48]. Nonetheless, reports indicate that some patients still cease to participate in their desired sports and experience subjective feelings of instability and fear of dislocations, which can potentially alter their perceptions of treatment success and have a significant impact on their overall knee‐related quality of life (QoL) [1, 17, 31, 42].

Patients' QoL has most commonly been assessed using various PROMs [32, 33, 34, 49]. Most often, such PROMs are reported as continuous data for a given cohort of patients (i.e., mean ± SD), which can be difficult to interpret and translate to an individual patient's response [38, 49]. Therefore, since most PROMs are evaluated in terms of statistical significance, which ultimately may not be perceived as important by the patients themselves, the minimal clinically important difference (MCID) was developed to aid in the overall understanding of these outcome measures but on a personal and patient‐relevant plane [9, 10, 11, 22, 23].

The goal of this study was to evaluate which of the demographic, clinical and/or pathoanatomical factors exert a predictive value for not reaching the MCID postoperatively when using the Banff Patellofemoral Instability Instrument 2.0 (BPII 2.0) as a validated disease‐specific QoL measure and function‐ and pain‐related PROMs in patients undergoing a tailored surgical approach for RLPD based on their individual risk profile.

Under the consideration of previously published data and clinical experience, it was hypothesized that patients with patellofemoral cartilage degeneration, increased BMI and/or a history of psychiatric disease would not reach the MCID following surgical treatment for RLPD [17, 21, 49, 52, 53].

## MATERIALS AND METHODS

This study received approval from the local ethics committee (Medical Council Baden‐Württemberg F‐2019‐070). This retrospective cohort study used a prospectively maintained database and included 237 consecutive patients (male/female 71/166; mean age 22.4 ± 6.8 years) with a history of RLPD who underwent a tailored surgical approach based on their clinical and anatomical risk factor profile.

To be eligible for inclusion in this study, patients had to meet the following criteria: (1) a history of ≥2 lateral patellar dislocations or (2) a first‐time patellar dislocation that required surgery following a period of at least 6 months of conservative treatment with persistency of symptoms. The following exclusion criteria were applied: (1) first‐time patellar dislocation that was specifically treated for an osteochondral flake fracture or an acute primary patellar stabilization procedure due to substantial injury to the medial ligament stabilizers; (2) previous MPFL‐R or previous bony procedures on the distal femur or proximal tibia, including tibial tuberosity osteotomy (TTO), trochleoplasty, varus‐producing and/or torsional distal femoral (DFO) and high tibial osteotomies (HTO); (3) patellofemoral pain without objective findings of RLPD; and (4) other previous ligamentous knee surgery (e.g., anterior cruciate ligament reconstruction; ACL‐R).

Preoperative MRI scans and routine radiographs (standing long‐leg axis radiographs and lateral view of the index knee in 30° of flexion radiographs) were obtained for each patient at the time of initial presentation and evaluated for the following parameters: degree of trochlear dysplasia, recorded as absent, low grade (Dejour type A), or high grade (Dejour type B‐D) [12]; patellar height using the Caton–Deschamps (CD) index, with ≥1.3 recorded as elevated [8]; tibial tuberosity–trochlear groove (TT‐TG) distance, with ≥16 mm recorded as elevated [4]; tibial tuberosity–posterior cruciate ligament (TT‐PCL) distance, with ≥25 mm recorded as elevated [3]; valgus malalignment was considered when the values of the mechanical hip‐knee‐ankle axis were ≥4° on standing long‐leg radiograph. Additionally, torsional MRI scans were performed in cases with clinically established rotational malalignment of the index leg (>70° of internal hip rotation in the prone position). Femoral torsion was calculated according to Murphy et al. [33], with >25° of femoral and >35° of tibial torsion recorded as increased [7, 52, 53]. Accordingly, trochleoplasty was indicated in patients exhibiting Dejour type B, C and/or D trochlear dysplasia; the consideration for concomitant tibial TTO was based on the following conditions: TT‐TG distance exceeding 16 mm, TT‐PCL distance surpassing 24 mm and/or CD index equal to or exceeding 1.3. Patients with femoral torsion exceeding 25° were evaluated for derotation DFO, whereas a valgus deformity equal to or greater than 4° led to the consideration of varus‐producing DFO. Postoperative protocols were standardized for all patients as previously published [52, 53].

Demographics (sex, age, height, weight and body mass index [BMI]), nicotine abuse, and the presence of psychiatric disease (including depression, anxiety and borderline personality disorders) were recorded for each patient at the time of the initial outpatient clinical examination. Evaluation of pathoanatomic risk factors, as well as patient disease‐specific QoL and PROMs for knee function and pain, were assessed and collected for each patient prior to surgery and at final follow‐up. The validated Banff Patellofemoral Instability Instrument 2.0 (BPII 2.0) was used to assess patient‐reported disease‐specific QoL [5], whereas knee joint function and patellofemoral pain were assessed using a numeric analogue scale (NAS pain/function) with values ranging from 0 to 10.

The cartilage status was evaluated using the semiquantitative ‘Area Measurement And Depth & Underlying Structures cartilage evaluation’ (AMADEUS) score system [24]. This score encompasses the assessment of three distinct parameters: the area of the cartilage defect, the morphology/depth of the cartilage defect, and the evaluation of underlying structures, including the presence of adjacent osseous defects and/or subchondral cysts, as well as bone marrow oedema–like lesions (BME). The cumulative score sums up to 100 points, ranging from 0 (indicating the worst condition) to 100 points (indicating the absence of osteochondral defects) [24].

The analysis of clinical and radiographic factors was conducted by one senior resident, one specialist and one senior surgeon specialized in patellofemoral surgery, with over 15 years of clinical experience. All surgical procedures were performed by the same senior surgeon and senior author of this study. Each patient underwent an individually tailored surgical treatment based on their clinical history, examination findings, diagnostic imaging and risk factor profile according to previously published recommendations [52, 53].

### Statistical analysis

The normality of the continuous data was assessed, and the data are presented as the means ± standard deviations (SDs; range). Categorical and dichotomous data are presented as frequency tabulations. The MCID was assessed using the distribution‐based method by calculating half of the SD of baseline BPII 2.0 score values [22].

The preliminary data analysis consisted of one‐way logistic regression for determining the ability of the individual variables to predict achieving MCID. Following the preliminary analysis, all variables that fulfilled the criteria of *p* ≤ 0.1 were entered into a conditional inference decision tree model to determine their optimal threshold in predicting whether the MCID will be achieved at the *p* ≤ 0.05 significance level (Tables 1 and 2). The recommended ‘rule of thumb’ for regression analysis is 10 patients per independent variable being assessed [37]. Having 17 predictor variables, the study population of *n* = 237 exceeded the recommended number of 10 × 17 = 170 patients; thus, the sample size was sufficient to perform the analyses [37]. This analysis was performed using R v.4.3.0 (R Core Team, www.R-project.org/).

**Table 1:** Analysis of each of the demographic, clinical and pathoanatomic parameters as predictive variables for reaching the BPII 2.0 MCID.

	Total (*n* = 237)	MCID <9.5 (*n* = 28)	MCID >9.5 (*n* = 209)	*p* Value
Variable	Mean ± SD	Mean ± SD	Mean ± SD	
Age (years)	22.4 ± 6.8	24.5 ± 7.6	22.1 ± 6.6	**0.013**
Sex (male/female)	71/166 (30/70%)	7/21 (25/75%)	64/145 (30.6/69.4%)	n.s.
Height (m)	1.7 ± 0.1	1.7 ± 0.1	1.7 ± 0.1	n.s.
Weight (kg)	71.9 ± 15.8	75.7 ± 14.9	71.4 ± 15.9	n.s.
BMI (kg/m^2^)	24.3 ± 4.7	25.9 ± 4.8	24.1 ± 4.6	n.s.
Nonsmoker/smoker	197/40 (81.1/16.9%)	24/4 (85.7/14.3%)	173/36 (82.8/17.2%)	n.s.
Psychiatric disease	9 (3.8%)	2 (7.1%)	7 (3%)	n.s.
Low‐/high‐grade TD	26/211 (11/89%)	3/25 (10.7/89.3)	23/186 (11/89%)	n.s.
CD index	1.2 ± 0.2	1.2 ± 0.2	1.2 ± 0.2	n.s.
TT‐TG (mm)	14.9 ± 4.5	14.8 ± 4.5	14.9 ± 4.5	n.s.
TT‐PCL (mm)	23.2 ± 3.8	23.4 ± 3.3	23.1 ± 3.9	n.s.
AMADEUS	82 ± 15.5	79.8 ± 19.7	82.3 ± 14.8	n.s.
Valgus alignment (°)	0.7 ± 3	1.7 ± 2.8	0.6 ± 3	n.s.
Follow‐up	34.9 ± 13.4	34.3 ± 13.1	35 ± 13.5	n.s.

*Note*: Bold value is statistically significant.

Abbreviations: AMADEUS, Area Measurement and Depth & Underlying Structures; BMI, body mass index; CD index, Caton–Deschamps index; MCID, minimal clinically important difference; TD, trochlear dysplasia; TT‐TG, tibial tubercle–trochlear groove; TT‐PCL, tibial tubercle–posterior cruciate ligament.

**Table 2:** Distribution of surgical treatment procedures.

Surgical procedure	Total (*n* = 237)		MCID <9.5 (*n* = 28)		MCID >9.5 (*n* = 209)		*p* Value
	*n*	(%)	*n*	(%)	*n*	(%)	
MPFL‐R	210	88.6	27	96.4	183	87.6	n.s.
TP	156	65.8	18	64.3	138	66	n.s.
TTO	74	31.2	6	21.4	68	32.5	n.s.
Varisation/rotational DFO	17	7.2	3	10.7	14	6.7	n.s.
Cartilage debridement	76	32.1	11	39.3	65	31.1	n.s.
Microdrilling	26	10.1	2	7.1	24	11.5	n.s.
Removal of loose bodies	31	13.1	5	17.9	26	12.4	n.s.
LR lengthening	158	66.7	19	67.9	139	66.5	n.s.
Medial reefing	16	6.7	1	3.6	15	7.2	n.s.

Abbreviations: DFO, distal femoral osteotomy; LR, lateral retinaculum; MCID, minimal clinically important difference; MPFL‐R, medial patellofemoral ligament reconstruction; TP, trochleoplasty; TTO, tibial tuberosity osteotomy.

## RESULTS

Evaluations were performed postoperatively at a mean follow‐up of 34.9 ± 13.4 months.

Patient characteristics, pathoanatomic factors and conducted surgical procedures are summarized in Tables 1 and 2. In the analysed cohort, the BPII 2.0 increased by an average of 36.1 points from a mean of 43.1 ± 20.9 points (3.2–56.9) preoperatively to 79.1 ± 18.3 points (12.7–87.8) postoperatively (*p* < 0.001). The NAS for knee function increased by an average of 3.8 from a mean of 4.6 ± 2.1 (0–8) preoperatively to 8.5 ± 1.3 (5–10) after surgery (*p* < 0.001), and the NAS for pain decreased by an average of 3.8 from 5.8 ± 2.4 (2–8) preoperatively to 2 ± 1.9 (0–10) postoperatively (*p* < 0.001).

The MCID was calculated at 9.5 points for the BPII 2.0. Out of the total cohort, 209 patients reached the threshold at final follow‐up, whereas 29 did not. The analysis of the individual variables' ability to predict achieving postoperative MCID revealed ‘preoperative function’ and ‘BPII 2.0’, as well as ‘age’ of the patient at the time of surgery, as significant variables (Tables 1 and 3). It was established that a score of 65.2 (*p* < 0.001) would be an optimal threshold for a preoperative BPII of 2.0, as 93.6% of patients had a preoperative BPII lower than the MCID. Furthermore, it was determined that in patients with a preoperative BPII 2.0 score value greater than 62.5, their age at the time of surgery should also be considered a predictive factor. Namely, 70.8% of the patients up to 24 years of age (*p* = 0.013) with a BPII 2.0 greater than 62.5 achieved the MCID, as opposed to only 21.4% of patients older than 24 years of age with a BPII 2.0 greater than 62.5.

**Table 3:** Analysis of each of the used PROMs with regard to reaching the BPII 2.0 MCID threshold.

	Total (*n* = 237)	MCID <9.5 (*n* = 28)	MCID >9.5 (*n* = 209)	*p* Value
Variable	Mean ± SD	Mean ± SD	Mean ± SD	
Preoperative pain NAS	5.8 ± 2.4	5.1 ± 2.6	5.9 ± 2.3	n.s.
Preoperative function NAS	4.6 ± 2.1	6.2 ± 2,1	4.4 ± 1.9	**0.05**
Preoperative BPII 2.0	43.1 ± 20.9	63.7 ± 22.9	40.3 ± 19.1	**<0.001**

*Note*: Bold values are statistically significant.

Abbreviations: BPII 2.0, Banff Patellofemoral Instability Instrument 2.0; MCID, minimal clinically important difference; NAS, numerical analogue scale; PROM, patient‐reported outcome measure.

Furthermore, when analysing preoperative function as a standalone parameter, the optimal threshold was calculated at a score value of 7 (*p* = 0.05), as 91% of patients with preoperative function lower than that achieved MCID, as opposed to 41.2% of patients with greater preoperative function, who did not. The type or extent of the surgical procedures, gender, BMI, psychiatric disorders, nicotine abuse and cartilage status did not exert a predictive value for reaching the calculated MCID threshold postoperatively (Tables 1 and 2).

Clinically, there were no cases of recurrence of LPD, whereas two patients had patellar subluxations at the final follow‐up. One patient had a low‐grade infection, which necessitated revision surgery, and no other significant complications were reported in any of the remaining included patients at the final follow‐up.

## DISCUSSION

The most important finding of this study was that the preoperative BPII 2.0 and the subjective rating of knee joint function provide the only predictive value for determining the probability of achieving the BPII 2.0 MCID threshold postoperatively in patients undergoing tailored surgical treatment for RLPD. In particular, the conducted analysis yielded that patients with BPII 2.0 score values >62.5 and subjective rating of knee function >7 (NAS) preoperatively have a significantly lower likelihood of achieving the MCID threshold after surgery. In addition, within the group of patients with higher BPII 2.0 values, age at the time of surgery appears to be an important factor, with patients older than 24 years of age having a lower likelihood of reaching the determined thresholds.

These results corroborate previously published findings that evaluated the MCID following various orthopaedic procedures (i.e., ACL‐R, knee cartilage therapy, hip arthroscopy and rotator cuff repair), demonstrating the trend that higher pathology‐specific PROM values are generally associated with a lower likelihood of reaching the MCID after surgical treatment [14, 23, 25, 32, 33, 49]. This trend can be perceived as intuitive, as patients with more severe symptoms and disease‐related impairment—resulting in lower preoperative PROM values—typically experience a more substantial potential for improvement across different facets that constitute their subjective perception of QoL. Consequently, they are more likely to exhibit a significant increase in their postoperative PROMs [15, 17, 34, 35, 36, 48]. In terms of age as a predictive factor, the presented result corroborates the findings of previous studies that showed that a higher age at the time of surgery was associated with a decline in postoperative outcomes, carrying a reduced odds ratio of achieving MCID following diverse orthopaedic interventions [9, 23, 35, 36].

Regarding RLPD treatment, however, few studies have examined the impact of predisposing factors on reaching MCID thresholds following procedures for patellar stabilization [17, 21, 48, 52, 53]. Walsh et al. [49] defined the MCID for various PROMs following MPFL‐R in a cohort of 139 patients and reported that older patients with higher preoperative PROM values were less likely to report improvement after surgery. Hiemstra et al. [17] investigated whether age at the time of surgery or sex can influence patient‐reported QoL and clinical outcomes after MPFL‐R in a cohort of 298 patients. The authors also used the BPII 2.0 score as the disease‐specific QoL measure and found that when adjusted for pathoanatomic risk factors, neither age at first dislocation nor sex influenced BPII 2.0 after MPFL‐R. However, age at the time of surgery did correlate with outcomes, with lower BPII 2.0 scores apparent for every 10‐year increase in age at the time of MPFL‐R. Conversely, Howells et al. [21] failed to demonstrate any correlation between the time from initial dislocation to surgery and multiple PROMs in a cohort of 201 patients treated with MPFL‐R using the gracilis tendon autograft. Among older patients, the higher impact of the underlying pathology on overall QoL could potentially be attributed to the enduring symptoms, resulting in over time exacerbating chronic pain and subjective instability. Additionally, age‐related factors might also contribute to an in‐time increase in cartilage damage and degenerative alterations in the patellofemoral joint due to chronic instability [17].

Cartilage lesions of the patellofemoral (PF) joint are frequently occurring comorbidities in patients with patellar instability, and it has been postulated that they are correlated with lower QoL and worse postoperative outcomes [18, 19, 42]. Interestingly, cartilage status evaluated according to AMADEUS score criteria did not exert a negative predictive influence on postoperative outcomes in the current analysis. Similarly, Holliday et al. [19] investigated whether postoperative disease‐specific QOL is affected by the presence and extent of PF cartilage lesions and found that even though 77.6% of 264 examined knees had Grade II–III PF cartilage lesions (according to International Cartilage Regeneration & Joint Preservation Society score [ICRS]), there was no correlation between the presence, size, or grade of lesions and BPII 2.0 scores preoperatively or at 12 or 24+ months following surgery. Likewise, Howells et al. [21] conducted a prospective analysis of clinical outcomes in 201 consecutive patients treated with MPFL‐R reconstruction using an autologous semitendinosus graft and found that the presence of patellofemoral articular cartilage damage at baseline was associated with poorer postoperative outcomes, but this did not reach statistical significance. Conversely, Žlak et al. [54] reported that worse preoperative PF cartilage status had negative effects on postoperative outcomes following isolated MPFL‐R and combined stabilization procedures in 161 knees. The negative predictive value of PF cartilage damage on postoperative outcomes was also reported in the study by Gonzalez et al. [14] in patients with ICRS Grade III or IV at the time of surgery (56% of 160 patient cohort) following isolated MPFL‐R at 4.8 years of follow‐up.

The BPII 2.0 presents a patient‐reported disease‐specific score designed to provide a detailed enquiry into patients' emotional and social well‐being, in addition to their physical symptoms and subjective assessment of the functional (dis)ability of the affected joint, thus establishing a comprehensive overview of all the elements that contribute to the QoL of patients with RLPD [5, 17, 18, 31]. In the analysed cohort, the preoperative BPII 2.0 score ranged from 3.2 to 56.9 (out of a maximal 100), corroborating the BPII 2.0 ranges reported in previous studies and underlining the generally reduced knee‐related QoL of affected patients [1, 4, 5, 52, 53]. Several factors have been outlined as significantly predisposing for lower values of BPII 2.0 scores in this population, including BMI, bilateral symptoms, age at first dislocation and severity of patellar maltracking assessed clinically by the J‐Sign [17, 18, 19, 31]. Interestingly, very little is still known about the effect of RLPD on the mental dimensions of patients' QoL [1, 6, 39, 46]. In this context, Balcarek et al. [1] demonstrated that untreated LPD significantly affected the physical dimension of patients' generic health‐related QoL (HRQoL), as well as their disease‐specific QoL as assessed through short form 36 (SF‐36) and BPII 2.0 scores. However, when compared to age‐equivalent normative data sets, the mental HRQoL dimension did not decrease before operative treatment, implying a good mental health status in the analysed cohort. Nevertheless, this dimension did exhibit an increase during the follow‐up period, indicating that treatment indeed impacts the mental health status of these patients [1]. However, the presented analysis did not find pre‐existing psychiatric diseases to be a predictive factor for reaching the MCID postoperatively.

Despite the predominantly favourable reported outcomes of surgical treatment of RLPD, foremost in terms of attaining patellar stability and restoration of joint function, in several studies, BPII 2.0 score values remained approximately 20 points less than a maximum value of 100 points [16, 17, 52, 53]. In the examined patient cohort, despite the absence of adverse events, few cases of instability recurrence and a significant increase in BPII score values, the results here also remained suboptimal, indicating that a reduced QoL is still present in a number of patients following these procedures. Even though it is unrealistic to anticipate a mean score value of 100 and considering the lack of normative data sets, findings do reinforce earlier conclusions that indicated the insufficiency of assessing the success of a treatment based solely on the presence or absence of recurrent patellar instability [5, 6, 16, 17]. For instance, regardless of the beneficial clinical outcomes of surgical treatment, certain individuals might still discontinue their desired athletic activities, contend with sensations of subjective instability, and grapple with a persistent fear of dislocation, which ultimately might alter their subjective perception of treatment success [1, 5, 6, 26, 30, 31, 46]. Nevertheless, given the lack of population‐based data on BPII 2.0 scores, the interpretation of postoperative data remains limited.

The influence of various anatomic factors on postoperative outcomes has been reported with varying results without a clear consensus in the available literature. (e.g., indication for concomitant TTO, trochleaplasty and derotational osteotomies) [2, 4, 20, 29, 30, 47]. Liu et al. [30] found no difference in the outcomes after MPFL‐R in patients with and without trochlear dysplasia. Conversely, Zimmermann et al. [52] reported that severe trochlear dysplasia, valgus malalignment of the affected leg and patella alta were directly associated with the failure of isolated MPFL‐R. Likewise, Hopper et al. [20] reported recurrence of patellar instability in all patients with severe trochlear dysplasia, whereas Zhang et al. [51] and Nelitz et al. [34] reported inferior outcome scores in patients with increased femoral torsion. In a recent study, Zimmermann et al. [52] showed that the correction of modifiable risk factors led to similarly good results as for isolated MPFL‐R in patients with mild forms of LPD considering the BPII 2.0 and subjective assessment of knee joint function. This corresponds to the findings of the current study in that the corrected pathoanatomy did not exert any predictive impact on postoperative outcome scores. Tanaka et al. [47] recently reported that normal thresholds of trochlear dysplasia vary based on the use of bony versus cartilaginous radiographic landmarks and that optimal cutoff values for almost all measurements differ between female and male patients. The authors concluded that these results potentially indicate the need for establishing sex‐specific parameters in the overall assessment of risk factors for patellofemoral instability [47].

The results of this study can serve as a valuable addition to the existing psychometric database for patellofemoral conditions. We posit that by considering the reported factors for not reaching the MCID postoperatively and integrating them with the previously established risk factors for unsatisfactory outcomes following patellar stabilising procedures, there is potential to reshape preoperative patient expectations, aid clinicians in patient counselling and enable informed and collaborative decision making in the treatment of this often very complex and multifactorial pathology.

Although this study presents a single‐centre, single‐surgeon perspective, including the results of a relatively high number of patients, the results must be interpreted within certain limitations. First to mention is the retrospective nature of the study design with its known limitations and potential bias characteristics. Additionally, despite the mean follow‐up of 34.95 ± 13.41 months, it remains possible that some patients might experience further improvement during even more extended follow‐up periods, which could potentially influence the MCID estimates. Furthermore, radiological analysis of femoral and tibial torsion was not conducted in all patients but only in those with clinically based suspicion of increased torsion. Furthermore, previous studies recommended that when evaluating the PROM after various orthopaedic procedures, at least one generic health status questionnaire should be included in addition to disease‐specific instruments to allow for a more comprehensive insight into the patients' QoL. However, Balcarek et al. [1] examined the mental and physical health‑related QoL in patients with RLPD and found a high correlation between the generic mental health outcomes measure and BPII 2.0 at both the preoperative and postoperative levels, indicating that BPII 2.0 encompasses a broad spectrum of QoL constructs in this patient population. Last, patient acceptable symptomatic state (PASS) and substantial clinical benefit (SCB) were not assessed for the current cohort. Although the MCID threshold is an acceptable and most commonly used method, there is also a need to report PASS and SCB to fully assess overall patient satisfaction [38].

## CONCLUSION

The results of this study indicate that the probability of reaching the BPII 2.0 MCID depends only on the preoperative disease‐specific QoL and subjective rating of knee joint function, as well as age at the time of surgery for patients undergoing tailored surgical treatment of RLPD. The results of this study can assist clinicians in advising and presenting patients with potential outcomes following treatment for this often complex and multifactorial pathology.

## AUTHOR CONTRIBUTIONS

Danko Dan Milinkovic: Data analysis, data interpretation, writing the paper and final approval. Sebastian Schmidt: Data acquisition, data interpretation and final approval. Julian Fluegel: Data acquisition, data interpretation and final approval. Sebastian Gebhardt: Data acquisition, data interpretation and final approval. Felix Zimmermann: Data acquisition, data interpretation and final approval. Peter Balcarek: Study design, data analysis, data interpretation, writing the paper and final approval.

## CONFLICT OF INTEREST STATEMENT

The authors declare no conflict of interest.

## ETHICS STATEMENT

This study was approved by the Ethics Committee of Baden‐Württemberg, Germany (F‐2019‐070).

## Data Availability

Raw data can be made available upon request. Data analysis protocols are listed in the ‘Statistical Analysis Supplement file’.
